# Differences in UV-C LED Inactivation of *Legionella* *pneumophila* Serogroups in Drinking Water

**DOI:** 10.3390/microorganisms10020352

**Published:** 2022-02-03

**Authors:** Helen Y. Buse, John S. Hall, Gary L. Hunter, James A. Goodrich

**Affiliations:** 1 US Environmental Protection Agency (USEPA), Office of Research and Development (ORD), Center for Environmental Solutions & Emergency Response (CESER), Homeland Security and Materials Management Division, Cincinnati, OH 45268, USA; hall.john@epa.gov (J.S.H.); goodrich.james@epa.gov (J.A.G.); 2 Black & Veatch, Greenville, SC 29607, USA; huntergl@bv.com

**Keywords:** potable water, premise plumbing, opportunistic pathogens, decontamination, treatment, UV-C LED disinfection, mechanisms

## Abstract

*Legionella pneumophila* (Lp) is an opportunistic pathogen that causes respiratory infections primarily through inhalation of contaminated aerosols. Lp can colonize premise plumbing systems due to favorable growth conditions (e.g., lower disinfectant residual, stagnation, warm temperatures). UV-C light-emitting diodes (UV-C LEDs) are an emerging water treatment technology and have been shown to effectively inactivate waterborne pathogens. In this study, the inactivation of four Lp strains (three clinical sg1, 4, and 6; and one sg1 drinking water (DW) isolate) was evaluated using a UV-C LED collimated beam at three wavelengths (255, 265, and 280 nm) and six fluence rates (0.5–34 mJ/cm^2^). Exposure to 255 nm resulted in higher log reductions at the lower fluences compared to exposures at 265 and 280 nm. Efficacy testing was also performed using a UV-C LED point-of-entry (POE) flow-through device. Based on the log inactivation curves, at 255 nm, the sg4 and sg6 clinical isolates were more susceptible to inactivation compared to the two sg1 isolates. However, at 265 and 280 nm, the sg1 and sg4 clinical isolates were more resistant to inactivation compared to the sg6 clinical and sg1 DW isolates. Differential log reductions were also observed using the POE device. Results indicate that although UV-C LED disinfection is effective, variations in Lp inactivation, wavelengths, and technology applications should be considered, especially when targeting specific isolates within premise plumbing systems.

## 1. Introduction

Legionellae are facultative intracellular Gram-negative bacteria [1]. Although they are commonly found in freshwater and soil environments [2,3], *Legionella* infection, or legionellosis, is mainly a result of exposure to engineered water systems due to favorable conditions enabling their growth and the generation of respirable aerosols for transmission [4,5,6]. There are currently over 60 known species within the *Legionella* genus, more than half of which have been associated with human disease [7,8]. *Legionella pneumophila* (Lp) is the most well-studied, since this species is responsible for >90% of legionellosis cases worldwide [9,10]. There are currently 15 known serogroup(sg)s of Lp with sg1 constituting the majority of Lp clinical isolates [9,10,11]. 

The prevalence of Lp in drinking water systems has been well documented, both in potable (e.g., building and residential systems) and non-potable (e.g., cooling tower) systems [12,13,14,15]. Legionellosis is a major public health concern due to drastically rising case numbers and incidence rates [16], but the disease is mostly preventable through the reduction in and elimination of the bacterium from engineered water systems [5]. Thus, effective disinfection strategies and treatment technologies are needed to prevent *Legionella* growth, human exposure, and subsequent infections in these systems. Current approaches and technologies to control *Legionella* include the use of chemical disinfectants (e.g., chlorine, monochloramine, chlorine dioxide) and various technologies such as copper-silver ionization, filtration, and ultraviolet (UV) treatment [17].

UV disinfection is a recognized and proven technology for decreasing waterborne disease risks from microbial pathogens in drinking water as its application does not alter the pH, result in the formation of disinfection byproducts or other harmful chemicals, or compromise water quality [18]. There are four regions in the UV spectrum: UV-A (315–400 nm), UV-B (280–315 nm), UV-C (200–280 nm), and vacuum UV (100–200 nm). UV-C light is considered the most germicidal since UV light absorption for DNA and RNA (200–300 nm) and proteins (185–320 nm) falls primarily in that range [19,20]. Exposure to this light range induces damage to nucleic acids and protein molecules, disrupting normal cellular processes, which results in microbial inactivation [21].

Mercury-based UV lamps are commonly used in drinking water and wastewater treatment plants [18]. Disinfection efficacy using low-pressure and medium-pressure mercury UV lamps has previously been reported for several *Legionella* species (e.g., *L. bozemanii*, *L. dumoffii*, *L. longbeacheae*, *L. micdadei*, and *L. pneumophila* (Lp)) as well as Lp sg1, 2, 7, and 8 strains [22,23,24,25,26,27,28,29,30]. However, there are major disadvantages of mercury-based UV lamps compared to UV light-emitting diodes (LEDs). LEDs can emit UV light at specific wavelengths and do not contain toxic materials or require a warmup time; thus, they can be cycled on and off more efficiently, are more compact and durable, and require less energy compared with mercury lamps [31].

The study of UV-C LED inactivation of *L. pneumophila* is very limited, especially within relevant drinking water test matrices and environments [32,33]. Thus, in this study, UV-C LED disinfection efficacy was evaluated for four Lp strains representing three clinically significant serogroups (sg1, 4, and 6) in drinking water utilizing both a collimated beam (at 255, 265, and 280 nm) and a point-of-entry (POE) treatment set-up (280 nm). Understanding the inactivation differences between Lp serogroups in drinking water, especially for UV-C LED POE applications, will help determine the most effective remediation strategies needed to target specific isolates during contamination events.

## 2. Materials and Methods

### 2.1. Bacterial Preparation

*Legionella pneumophila* sg 1 Philadelphia-1 strain (sg1) and *L. pneumophila* serogroup 4 Los Angeles-1 strain (sg4) are both clinical isolates derived from the lungs of a pneumonia patient (American Type Culture Collection, Manassas, VA USA (ATCC); ATCC strain 33152 and 33156, respectively). *L. pneumophila* serogroup 6 Chicago-2 strain (sg6) is also a clinical isolate derived from a lung biopsy (ATCC 33215). The *L. pneumophila* sg 1 drinking water strain (sg1 DW) was isolated during a previous study [12]. *L. pneumophila* (Lp) cells were grown and enumerated as previously described [34]. Briefly, frozen stock cultures were thawed and streaked onto buffered charcoal yeast extract (BCYE) agar plates (BD Diagnostics, Franklin Lakes, NJ, USA) and incubated at 37 °C for 72 h. An Lp colony was inoculated into 10 mL buffered yeast extract (BYE) broth and grown overnight (ON) with continuous shaking at 37 °C. ON cultures were centrifuged (2420 rcf, 10 min, room temperature) and washed three times with 10 mL dfH_2_O (UV-light dechlorinated, 0.22 μm filtered drinking water).

### 2.2. Culture Analyses

Lp densities, as measured by colony-forming units (CFU), were determined by spread plating undiluted and serially diluted bacterial suspensions on BCYE plates incubated for 3–5 d at 37 °C. Heterotrophic plate count (HPC) bacteria were enumerated by the spread plate method on Reasoner’s 2A agar (R2A, Difco Laboratories, Detroit, MI, USA) following incubation at 28 °C for 7 d. The limit of detection (LOD) for water samples was 0.7 log_10_ CFU/mL.

### 2.3. Water Quality Measurements

Free and total chlorine were measured using the N,N-diethyl-p-phenylenediamine (DPD) Method (Pocket ColorimeterTM II, Hach, Loveland, CO, USA); pH and temperature (ExTech 407227, Extech Instruments, Nashua, NH, USA); hardness (ethylenediaminetetraacetic acid (EDTA) Titration Method, Hach, Loveland, CO, USA); turbidity (2100 Q Portable Turbidimeter, Hach, Loveland, CO, USA); ferrous and total iron (1, 10 phenanthroline Method, Hach, Loveland, CO, USA); and absorbance and ultraviolet transmittance (UVT) of the test water at 255, 265, and 280 nm (DR6000 UV-VIS spectrophotometer, Hach, Loveland, CO, USA).

### 2.4. Collimated Beam Tests

UV-C LED treatment tests were performed at three wavelengths using a collimated-beam device (PearlLab Beam T 255/265/280, AquiSense Technologies, Erlanger, KY, USA) using Lp cells suspended in dfH_2_O. The UV-C LED collimated beam was fixed at 20 mm above the surface of the sample (25 mL of 10^6^ CFU/mL prepared *L. pneumophila* culture as described above) contained in a 100 × 15 mm Petri dish (sample depth of 3.2 mm). The UV transmittance (UVT) of dfH_2_O was measured at 255, 265, and 280 nm, and the peaks were observed at 80%, 87%, and 92%, respectively.

Fluence is typically dependent on the characteristics of the treated water and the design and operation of the UV disinfection device. In this study, to achieve UV fluence of 0, 0.5, 1, 2, 5, and 10 mJ/cm^2^, exposure time was controlled to 0, 17, 34, 67, 168, and 336 s at 255 nm; 0, 4, 9, 18, 44, and 89 s at 265 nm; and 0, 2, 3, 6, 15, and 31 s at 280 nm, respectively. The additional UV fluences of 16 and 34 mJ/cm^2^ were evaluated at 280 nm with exposure times of 49 and 105 s, respectively. All experiments were conducted at room temperature under a biological safety cabinet with three replicates for each strain and fluence. After UV-C LED exposure, culture analysis was performed on the samples as described above.

### 2.5. Point-of-Entry (POE) Treatment Design and Sample Collection

**Figure 1:**
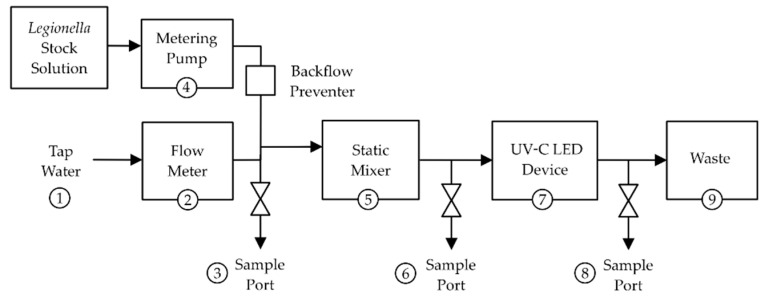
Graphical depiction of the UV-C LED POE device test set-up. (1) Source of drinking water delivered via tygon tubing, (2) flow meter, (3) influent sampling port, (4) pump used to deliver the Lp inoculum into the water with a backflow preventer (tube clamp), (5) static mixer, (6) pre-treatment sampling port, (7) UV-C LED POE device, (8) post-treatment sampling port, (9) 55-gallon biological liquid waste collector. See Supplementary Figure S1 for a schematic and image of the testing set-up.

Disinfection tests under drinking water flow conditions were performed using a commercial UV-C LED 280 nm POE residential system (PearlAqua Deca, AquiSense Technologies, Erlanger, KY, USA). Based on the product manual, the UV fluence was estimated at 30–40 mJ/cm^2^ at a 19–25 liter per min (lpm) or 5.0–6.5 gallon per minute (gpm), flow rate. The manufacturer’s water quality specifications for optimal performance of the unit are ≥90% UV transmittance, ≤10 µm particulate size, ≤120 mg/L CaCO_3_ hardness, and ≤0.3 mg/L iron. The UVT at 280 nm of the drinking water (% mean ± standard deviation) was 98.3 ± 3.0, respectively. To allow measurements of Lp levels before and after treatment as well as to monitor influent water quality, a specialized set-up of the POE device was constructed under a biological safety cabinet (Figure 1).

The influent drinking water was local tap water from the laboratory sink faucet. Water samples were collected in sterile 1 L plastic bottles containing 1 mL of 10% *w*/*v* sodium thiosulfate to neutralize secondary disinfectant residual, except for samples collected for water quality analyses.

For each experimental replicate, the influent drinking water tap was turned on followed by the POE device and pump; the latter was set at a predetermined flow rate to feed the concentrated Lp inoculum to achieve the desired test concentration. The tube clamp was then manually released, and the system was allowed to run for 5 s before collecting 1 L at the pre-treatment port, followed by another 1 L collection at the post-treatment port. After sample collection, the tube clamp was reengaged, and both the pump and the POE device were turned off. The sampling ports were then all flushed, and the tap water was turned off. There were five experimental replicates performed for each Lp strain with this set-up. All experiments were conducted at room temperature (20 ± 2 °C). Culture analyses were performed on the influent, pre-, and post-treatment samples as described above.

### 2.6. Statistical Analyses

A two-way analysis of variance (ANOVA), using the Tukey multiple comparisons test, was conducted between each strain and wavelength. *p* values < 0.05 were considered statistically significant. Analyses were performed and graphs generated using Prism 8 (GraphPad Software, San Diego, California, CA, USA). The measure of uncertainty for the log reductions following the POE device treatment (standard deviation (SD_LR_)) was calculated using the following Equation (1) [35]:

SD_LR_ = [(SD^2^ _Con_ / n_Con_) + (SD^2^ _Tr_ / n_Tr_)]^½^ (1)

where SD_Con_ and SD_Tr_ are the standard deviations of the log reduction values for the untreated controls and treated samples, respectively, and n_Con_ and n_Tr_ are the numbers of samples for the control and treated samples, respectively.

## 3. Results and Discussion

### 3.1. Collimated Beam Tests

**Figure 2:**
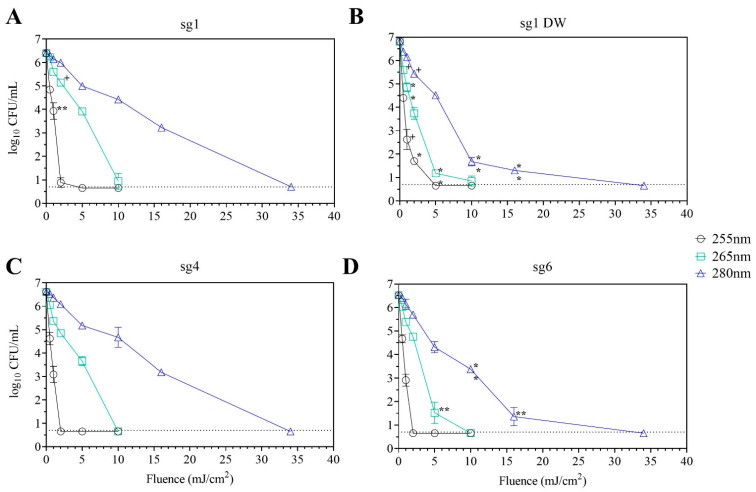
Inactivation of Lp strains using the UV-C LED collimated beam. Lp sg 1 (**A**), sg 1 DW isolate (**B**), sg 4 (**C**), and sg 6 (**D**) were exposed to various UV fluences at 255 nm (black open circles), 265 nm (green squares), and 280 (blue triangles) as described in Materials and Methods. Data (mean ± standard deviation) are representative of three replicates for each strain. The limit of detection of 0.7 log_10_ CFU/mL is indicated by the dotted line. Statistical significance when compared to all other strains are denoted as ^+^ for *p* < 0.05, * for *p* < 0.01, and ** for *p* < 0.001. See also Supplemental Materials Table S1.

At 255 nm, there were 1.6–2.3 log_10_ CFU decreases at 0.5 mJ/cm^2^ and 2.5–4.1 log_10_ decreases at 1 mJ/cm^2^ for each of the four strains (Figure 2, black lines; see also Supplemental Materials Table S1). The sg1 drinking water (DW) displayed the highest log reductions at 0.5 and 1 mJ/cm^2^ compared to the other strains. However, statistically significant levels of 1.7 log_10_ CFU/mL were detectable for the sg1 DW strain at 2 mJ/cm^2^ compared to below the limit of detection (LOD) for sg4 and sg6 and about 1 log_10_ CFU/mL for the sg1 clinical strain (Figure 2B, black line, indicated with *, *p* < 0.01). This suggests that at 255 nm, the sg1 DW strain was more resistant to UV-C LED disinfection compared to the other strains, but at 5 mJ/cm^2^, no culturable cells were detected for all strains.

In contrast, there were detectable CFU levels during 265 nm exposure at 2 and 5 mJ/cm^2^ with 1.2–3.0 and 2.4–6.0 log reductions, respectively, for all strains (Figure 2, green lines). There were statistically significant differences between the sg1 and sg4 strains indicating less susceptibility to inactivation compared to the sg1 DW and sg6 strains at 5 mJ/cm^2^ (Figure 2B,D, green line, indicated with ** *p* < 0.001). A similar trend was observed during 280 nm exposure where the sg1 and sg4 strains were statistically less susceptible to inactivation compared to the sg1 DW and sg6 strains at higher fluences of 10 and 16 mJ/cm^2^ (Figure 2B,D, blue line, indicated with **, *p* < 0.001).

Other studies have evaluated Lp inactivation using UV-C LED collimated beam devices at similar UV-C wavelengths of 254–256, 265–268.6, and 280–288.6 nm [32,33]. For Lp inactivation comparisons between studies (this study vs. Grossi et al. [32] vs. Rattanakul et al. [33]) at 254–256 nm, approximately three log reductions were observed at 1 vs. 8 vs. 5 mJ/cm^2^; at 265–268 nm, 4 vs. 6 vs. 3 mJ/cm^2^; and at 280–288.6 nm, 16 vs. 14 vs. 7 mJ/cm^2^. These differences could be attributed to variations in the test medium and use of other Lp strains (e.g., Grossi et al. evaluated the same sg1 clinical strain used in this study but suspended in 0.85% saline solution and not with dechlorinated, filtered tap water as in this study; while Rattanakul et al. utilized an Lp isolate of unknown serogroup and source suspended in phosphate-buffered saline).

Moreover, in one study, Lp inactivation was observed to be the highest at 265 nm. This was hypothesized to be due to the maximum absorbance of DNA at 260 nm, resulting in peak UV-induced DNA damage [33]. However, that trend was not observed in this study nor in the second study [32], indicating that other mechanisms of UV-induced cellular damage may contribute to the inactivation of *L. pneumophila* at these wavelengths. The observed variations between studies underscore the need for systematic testing of different *L. pneumophila* strains to understand the efficacy and mechanisms of UV-C LED treatment. This is especially warranted since UV LED technology is increasingly applied in drinking water treatment to address the public health concern of *L. pneumophila* and other waterborne pathogens in these systems. 

In this study, 255 nm of exposure resulted in the most rapid log reductions at the lower fluences versus the 265 and 280 nm wavelengths. Under 0.5 fluence at 255 nm, between 1.6–2.3 log reductions were observed for each Lp strain, where similar log reductions were observed under 2–5 fluence at 265 nm and 5–16 fluence at 280 nm. Lp cells were exposed for 17 s at 255 nm to achieve 0.5 fluence, 18–44 s at 265 nm to achieve 2–5 fluence, and 15–49 s at 280 nm to achieve 5–16 fluence. Thus, the rapid log reductions observed at 255 nm may be due to longer exposure times and different inactivation mechanisms resulting from this wavelength exposure.

### 3.2. Point-of-Entry Tests

For the 280 nm wavelength, the additional 16 and 34 mJ/cm^2^ fluences were added to the collimated beam test for comparisons to Lp inactivation using a point-of-entry (POE) UV-C LED treatment device. At the tap water flow rates of the POE test set-up (2.5–3.0 gpm, Figure 1), it was estimated that UV-C LED exposure at 280 nm within the POE device would be in the 16–34 mJ/cm^2^ fluence range. Table 1 shows the influent drinking water quality parameters for each experimental run, which are typical of the drinking water source used in this study [12,34].

**Figure 3:**
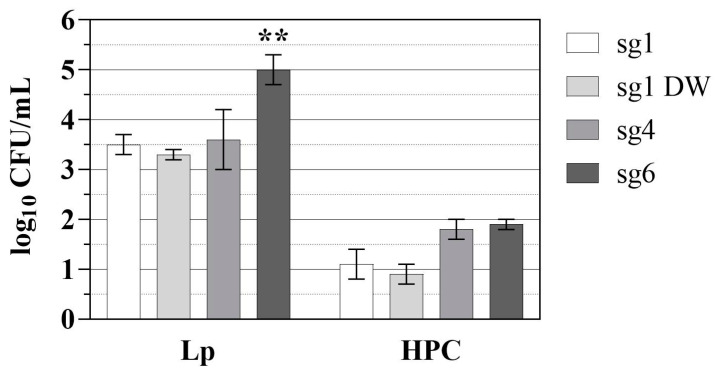
Lp and HPC log reductions using the UV-C LED POE device. Data represent the mean ± SD_LR_ log_10_ CFU mL^−1^ reductions in Lp sg 1 (white bars), sg 1 DW isolate (light grey bars), sg 4 (dark grey bars), and sg 6 (black bars) and the reduction in HPC levels in the drinking water source during each of the Lp strain experimental runs. SD_LR_ was calculated as described in Section 2.6. Data are representative of five replicates for each strain. Statistical significance when compared to all other strains is denoted as ** for *p* < 0.001. See Supplemental Materials Table S2.

Figure 3 displays the log reductions of Lp and heterotrophic plate counts (HPC) calculated by subtracting the post-UV-treated CFU levels from the pre-treatment levels. Based on the collimated beam experiments at 280 nm, the sg1 DW and sg6 strains were expected to be more susceptible to inactivation compared to the sg1 and sg4 strains (Figure 2, blue line). However, for the POE tests, sg6 displayed the highest reduction (mean ± SD_LR_) of 5.0 ± 0.3 log_10_ CFU/mL compared to 3.5 ± 0.2, 3.3 ± 0.1, and 3.6 ± 0.6 log_10_ CFU/mL for sg1, sg1 DW, and sg4, respectively (Figure 3, *p* < 0.001; see also Supplemental Materials Table S2).

The differences in inactivation observed with the collimated beam at 280 nm (Figure 2, blue lines) and the POE device (Figure 3) suggest that different UV exposure conditions (e.g., static versus flowing suspensions) and the suspension buffer (filtered, dechlorinated water versus tap water) can impact UV inactivation. Suspended particles in drinking water (e.g., mineral and metal oxides, clay) can protect microbial cells from UV exposure by shielding, blocking, scattering, or absorbing UV light and negatively impact UV disinfection efficacy [18,36,37,38]. Thus, another consideration for suspended particles would be the impact of microplastics on UV treatment. Microplastics are accumulating globally at an alarming rate and can be found in almost every environment and organism [39]. Concentrations of microplastics in drinking water can be as high as 9.2 particles/L and in bottled water, 5.4 × 10^7^ particles/L [40]. Notably, microplastics have been found to negatively impact UV and chlorine disinfection during drinking and wastewater treatment [41] and can accumulate biofilm material on their surfaces [42]. Thus, further considerations should be given to this emerging contaminant of concern, especially during the evaluation of new drinking water treatment technologies.

Previous studies have shown *Legionella* levels in finished water and the distribution system to be low, with <4% of samples being *Legionella* PCR-positive (3.5 log_10_ target copies/L) and < 1% of samples containing culturable Lp (1 MPN/100 mL), respectively [43,44]. The UV-C LED treatment resulted in > 3 log_10_ CFU/mL reduction in each Lp strain (Figure 3), indicating the effectiveness of the POE device for Lp disinfection. Additionally, Hall et al. reported *Legionella* culture-negative water samples and the absence of *Legionella* infections in a hospital building for 13 years after the installation of a UV-POE device [45]. In the same study, drinking water samples in neighboring facilities were *Legionella* culture-positive, further emphasizing the use of POE treatment as an effective potential strategy for controlling the occurrence of opportunistic pathogens in premise plumbing systems.

There were no statistical differences between HPC log reductions for each of the Lp experimental runs (Figure 3). The mean ± SD_LR_ log HPC reductions for the sg1, sg1 DW, sg4, and sg6 strains were 1.1 ± 0.3, 0.9 ± 0.2, 1.8 ± 0.2, and 1.9 ± 0.1, respectively. HPC microorganisms recovered from drinking water are typically non-pathogenic, do not pose any increased health risk, and are inadequate surrogates for pathogenic bacteria; however, HPC can be useful for validating and verifying water treatment processes and indicating conditions supportive of microbial regrowth [46,47]. Interestingly, previous studies have indicated UV-C LED treatment can select for UV-resistant subsets of HPC populations (e.g., *Methylorubrum* spp. (formerly *Methylobacterium*) [48]) and *Brevibacillus* spp.), but it is unclear whether that selection poses any public health concerns or negative impacts on water quality [49,50].

There are an increasing number of studies evaluating non-culture-based methods to access the viable but non-culturable (VBNC) status of UV-inactivated pathogens, such as adenosine triphosphate (ATP) measurements, flow cytometry, and quantitative PCR [51,52]. When exposed to low-nutrient or other stressful environments, the transition into the VBNC state is regarded as a microbial survival mechanism; thus, when conditions become favorable (e.g., post-disinfectant treatment), there is resuscitation that may pose a public health concern [53]. Moreover, advantageous DNA damage responses and repair activities in pathogenic bacteria can result in a decreased susceptibility to UV treatment [54], but those processes are still unclear for *L. pneumophila* [55]. Thus, the mechanisms of UV inactivation warrant further examination to understand the long-term effects on microbial populations post-UV treatment of drinking water.

## 4. Conclusions

The efficacy of UV-C LED inactivation can differ, even between strains of the same species. Understanding these differences can be useful, especially in cases where an environmental source is linked to a clinical case and an effective remediation strategy is needed to target that specific isolate within drinking water systems. Knowing how pathogen characteristics such as outer membrane properties dictate inactivation efficacy is important for effective remediation. Although different reduction levels were observed for each Lp strain at 255, 265, and 280 nm, complete inactivation was achieved quickly. Moreover, by simulating high contaminant loads, the POE UV-C LED device demonstrated at least between a 3-log and 5-log reduction in *Legionella pneumophila*.

Further work is needed to optimize the operating conditions and set-up of this POE device and to examine other UV-C LED technologies. In addition to aiming to achieve higher inactivation levels, biofilm- or particle-associated forms of pathogens should also be tested as they may be inactivated differently or less efficiently than their evenly suspended forms.

## Figures and Tables

**Table 1:** Influent water quality parameters for each experimental run.

Parameter (Units)	*Legionella pneumophila* Strain Used			
	sg1	sg1 DW	sg4	sg6
pH	8.60 ± 0.05	8.70 ± 0.04	9.0 ± 0.09	9.1 ± 0.01
Temperature (°C)	9.9 ± 0.1	15.1 ± 0.1	19.9 ± 0.1	17.1 ± 0.1
Hardness (mg/L CaCO_3_)	130 ± 14	130 ± 14	120 ± 0	140 ± 0
Turbidity (NTU)	0.25 ± 0.10	0.33 ± 0.00	0.64 ± 0.02	0.22 ± 0.00
Free Chlorine (mg/L) Total Chlorine (mg/L)	0.93 ± 0.00	0.92 ± 0.00	0.73 ± 0.00	0.92 ± 0.01
	1.04 ± 0.00	1.04 ± 0.01	0.85 ± 0.01	1.05 ± 0.03
Ferrous Iron (mg/L) Total Iron (mg/L)	0.00 ± 0.00	0.01 ± 0.00	0.00 ± 0.00	0.00 ± 0.00
	0.05 ± 0.00	0.04 ± 0.01	0.00 ± 0.00	0.01 ± 0.00

Data are presented as mean ± standard deviation of duplicate readings for each parameter.

## Supplementary Materials

The following are available online at https://www.mdpi.com/article/10.3390/microorganisms10020352/s1, Figure S1: Schematic and image of the UV-C LED POE device test set-up, Table S1: CFU levels of Lp strains after UV-C LED collimated beam exposure, and Table S2: CFU levels pre- and post-UV-C LED POE treatment.

## References

[1] Fields B.S., Bitton G. (2003). Legionellae. Encyclopedia of Environmental Microbiology.

[2] Fliermans C.B., Cherry W.B., Orrison L.H., Smith S.J., Tison D.L., Pope D.H. (1981). Ecological distribution of *Legionella pneumophila*. Appl. Environ. Microbiol..

[3] Travis T.C., Brown E.W., Peruski L.F., Siludjai D., Jorakate P., Salika P., Yang G., Kozak N.A., Kodani M., Warner A.K. (2012). Survey of *Legionella* species found in Thai soil. Int. J. Microbiol..

[4] Buse H.Y., Schoen M.E., Ashbolt N.J. (2012). Legionellae in engineered systems and use of quantitative microbial risk assessment to predict exposure. Water Res..

[5] Garrison L.E., Kunz J.M., Cooley L.A., Moore M.R., Lucas C., Schrag S., Sarisky J., Whitney C.G. (2016). Vital Signs: Deficiencies in Environmental Control Identified in Outbreaks of Legionnaires’ Disease—North America, 2000–2014. MMWR Morb. Mortal. Wkly. Rep..

[6] Nisar M.A., Ross K.E., Brown M.H., Bentham R., Whiley H. (2020). Water stagnation and flow obstruction reduces the quality of potable water and increases the risk of legionelloses. Front. Environ. Sci..

[7] Lindsay D.S., Brown A.W., Brown D.J., Pravinkumar S.J., Anderson E., Edwards G.F. (2012). *Legionella longbeachae* serogroup 1 infections linked to potting compost. J. Med. Microbiol..

[8] Mondino S., Schmidt S., Rolando M., Escoll P., Gomez-Valero L., Buchrieser C. (2020). Legionnaires’ Disease: State of the Art Knowledge of Pathogenesis Mechanisms of *Legionella*. Annu. Rev. Pathol..

[9] Beauté J. (2017). Legionnaires’ disease in Europe, 2011 to 2015. Eurosurveillance.

[10] Yu V.L., Plouffe J.F., Pastoris M.C., Stout J.E., Schousboe M., Widmer A., Summersgill J., File T., Heath C.M., Paterson D.L. (2002). Distribution of *Legionella* species and serogroups isolated by culture in patients with sporadic community-acquired legionellosis: An international collaborative survey. J. Infect. Dis..

[11] Lück C., Fry N., Helbig J., Jarraud S., Harrison T., Buchrieser C., Hilbi H. (2013). Typing Methods for *Legionella*. Legionella.

[12] Buse H.Y., Morris B.J., Gomez-Alvarez V., Szabo J.G., Hall J.S. (2020). *Legionella* diversity and spatiotemporal variation in the occurrence of opportunistic pathogens within a large building water system. Pathogens.

[13] Donohue M.J., King D., Pfaller S., Mistry J.H. (2019). The sporadic nature of *Legionella pneumophila*, *Legionella pneumophila* Sg1 and *Mycobacterium avium* occurrence within residences and office buildings across 36 states in the United States. J. Appl. Microbiol..

[14] Llewellyn A.C., Lucas C.E., Roberts S.E., Brown E.W., Nayak B.S., Raphael B.H., Winchell J.M. (2017). Distribution of *Legionella* and bacterial community composition among regionally diverse US cooling towers. PLoS ONE.

[15] Völker S., Schreiber C., Kistemann T. (2010). Drinking water quality in household supply infrastructure—A survey of the current situation in Germany. Int. J. Hyg. Environ. Health.

[16] Centers for Disease Control and Prevention (CDC) National Notifiable Diseases Surveillance System (NNDSS). https://www.cdc.gov/nndss/index.html.

[17] US Environmental Protection Agency (USEPA) (2016). Technologies for Legionella Control in Premise Plumbing Systems: Scientific Literature Review.

[18] US Environmental Protection Agency (USEPA) (2006). UV Disinfection Guidance Manual for the Final LT2ESWTR.

[19] Beck S.E., Rodriguez R.A., Hawkins M.A., Hargy T.M., Larason T.C., Linden K.G. (2015). Comparison of UV-induced inactivation and RNA damage in MS2 phage across the germicidal UV spectrum. Appl. Environ. Microbiol..

[20] Prasad S., Mandal I., Singh S., Paul A., Mandal B., Venkatramani R., Swaminathan R. (2017). Near UV-Visible electronic absorption originating from charged amino acids in a monomeric protein. Chem. Sci..

[21] Hijnen W.A., Beerendonk E.F., Medema G.J. (2006). Inactivation credit of UV radiation for viruses, bacteria and protozoan (oo)cysts in water: A review. Water Res..

[22] Antopol S.C., Ellner P.D. (1979). Susceptibility of *Legionella pneumophila* to ultraviolet radiation. Appl. Environ. Microbiol..

[23] Cervero-Aragó S., Sommer R., Araujo R.M. (2014). Effect of UV irradiation (253.7 nm) on free *Legionella* and *Legionella* associated with its amoebae hosts. Water Res..

[24] Gilpin R.W., Dillon S.B., Keyser P., Androkites A., Berube M., Carpendale N., Skorina J., Hurley J., Kaplan A.M. (1985). Disinfection of circulating water systems by ultraviolet light and halogenation. Water Res..

[25] Knudson G.B. (1985). Photoreactivation of UV-irradiated *Legionella pneumophila* and other *Legionella* species. Appl. Environ. Microb..

[26] Miyamoto M., Yamaguchi Y., Sasatsu M. (2000). Disinfectant effects of hot water, ultraviolet light, silver ions and chlorine on strains of *Legionella* and nontuberculous mycobacteria. Microbios.

[27] Muraca P., Stout J.E., Yu V.L. (1987). Comparative assessment of chlorine, heat, ozone, and UV light for killing *Legionella pneumophila* within a model plumbing system. Appl. Environ. Microbiol..

[28] Oguma K., Katayama H., Ohgaki S. (2004). Photoreactivation of *Legionella pneumophila* after inactivation by low- or medium-pressure ultraviolet lamp. Water Res..

[29] Schmid J., Hoenes K., Rath M., Vatter P., Hessling M. (2017). UV-C inactivation of *Legionella rubrilucens*. GMS Hyg. Infect. Control.

[30] Wilson B., Roessler P., Van Dellen E., Abbaszadegan M., Gerba C.P. Coliphage MS-2 as a UV Water Disinfection Efficacy Test Surrogate for Bacterial and Viral Pathogens. Proceedings of the AWWA Water Quality Technology Conference.

[31] Carlson K., Boczek L., Chae S., Ryu H. (2020). Legionellosis and recent advances in technologies for *Legionella* control in premise plumbing systems: A review. Water.

[32] Grossi M.R., Dey R., Ashbolt N.J. (2018). Searching for activity markers that approximate (VBNC) *Legionella pneumophila* infectivity in amoeba after ultraviolet (UV) irradiation. Water.

[33] Rattanakul S., Oguma K. (2018). Inactivation kinetics and efficiencies of UV-LEDs against *Pseudomonas aeruginosa*, *Legionella pneumophila*, and surrogate microorganisms. Water Res..

[34] Buse H.Y., Morris B.J., Struewing I.T., Szabo J.G. (2019). Chlorine and monochloramine disinfection of *Legionella pneumophila* colonizing copper and polyvinyl chloride drinking water biofilms. Appl. Environ. Microbiol..

[35] Zelver N., Hamilton M., Goeres D., Heersink J., Doyle R.J. (2001). Development of a Standardized Antibiofilm Test. Methods in Enzymology.

[36] Christensen J., Linden K.G. (2003). How particles affect UV light in the UV disinfection of unfiltered drinking water. J. Am. Water Work. Assoc..

[37] Qualls R.G., Flynn M.P., Johnson J.D. (1983). The role of suspended particles in ultraviolet disinfection. J. Water Pollut. Control Fed..

[38] Wu Y., Clevenger T., Deng B. (2005). Impacts of goethite particles on UV disinfection of drinking water. Appl. Environ. Microbiol..

[39] Stubbins A., Law K.L., Muñoz S.E., Bianchi T.S., Zhu L. (2021). Plastics in the Earth system. Science.

[40] Zhang Q., Xu E.G., Li J., Chen Q., Ma L., Zeng E.Y., Shi H. (2020). A review of microplastics in table salt, drinking water, and air: Direct human exposure. Environ. Sci. Technol..

[41] Shen M., Zeng Z., Li L., Song B., Zhou C., Zeng G., Zhang Y., Xiao R. (2021). Microplastics act as an important protective umbrella for bacteria during water/wastewater disinfection. J. Clean. Prod..

[42] Wright R.J., Erni-Cassola G., Zadjelovic V., Latva M., Christie-Oleza J.A. (2020). Marine plastic debris: A new surface for microbial colonization. Environ. Sci. Technol..

[43] LeChevallier M.W. (2019). Monitoring distribution systems for *Legionella pneumophila* using Legiolert. AWWA Water Sci..

[44] King D.N., Donohue M.J., Vesper S.J., Villegas E.N., Ware M.W., Vogel M., Furlong E.F., Kolpin D.W., Glassmeyer S., Pfaller S. (2016). Microbial pathogens in source and treated waters from drinking water treatment plants in the United States and implications for human health. Sci. Total Environ..

[45] Hall K.K., Giannetta E.T., Getchell-White S.I., Durbin L.J., Farr B.M. (2003). Ultraviolet light disinfection of hospital water for preventing nosocomial *Legionella infection*: A 13-year follow-up. Infect. Control Hosp. Epidemiol..

[46] Allen M.J., Edberg S.C., Reasoner D.J. (2004). Heterotrophic plate count bacteria—What is their significance in drinking water?. Int. J. Food Microbiol..

[47] Bartram J., Cotruvo J., Exner M., Fricker C.A.G., World Health Organization (WHO) (2003). Heterotrophic Plate Counts and Drinking-Water Safety the Significance of HPCs for Water Quality and Human Health.

[48] Green P.N., Ardley J.K. (2018). Review of the genus *Methylobacterium* and closely related organisms: A proposal that some *Methylobacterium* species be reclassified into a new genus, *Methylorubrum* gen. nov. Int. J. Syst. Evol. Microbiol..

[49] Mariita R.M., Blumenstein S.A., Beckert C.M., Gombas T., Randive R.V. (2021). Disinfection performance of a drinking water bottle system with a UV subtype C LED cap against waterborne pathogens and heterotrophic contaminants. Front. Microbiol..

[50] Oguma K., Kanazawa K., Kasuga I., Takizawa S. (2018). Effects of UV irradiation by light emitting diodes on heterotrophic bacteria in tap water. Photochem. Photobiol..

[51] Kong X., Ma J., Wen G., Wei Y. (2016). Considerable discrepancies among HPC, ATP, and FCM detection methods in evaluating the disinfection efficiency of Gram-positive and -negative bacterium by ultraviolet radiation and chlorination. Desalination Water Treat..

[52] Yang C., Sun W., Ao X. (2020). Bacterial inactivation, DNA damage, and faster ATP degradation induced by ultraviolet disinfection. Front. Environ. Sci. Eng..

[53] Pinto D., Santos M.A., Chambel L. (2015). Thirty years of viable but nonculturable state research: Unsolved molecular mechanisms. Crit. Rev. Microbiol..

[54] Podlesek Z., Žgur Bertok D. (2020). The DNA damage inducible SOS response is a key player in the generation of bacterial persister cells and population wide tolerance. Front. Microbiol..

[55] Charpentier X., Kay E., Schneider D., Shuman H.A. (2011). Antibiotics and UV radiation induce competence for natural transformation in *Legionella pneumophila*. J. Bacteriol..

