# Supplementary material for: Differences in UV-C LED Inactivation of Legionella pneumophila Serogroups in Drinking Water
Source: Microorganisms. 2022 Feb 3;10(2):352. doi: 10.3390/microorganisms10020352 (PMC8877565; doi:10.3390/microorganisms10020352)
Supplement: Supplementary file 1 [file microorganisms-10-00352-s001.zip › microorganisms-1571811-supplementary.pdf]

*microorganisms*

*Supplementary Materials*

# Differences in UV-C LED Inactivation of *Legionella pneumophila* Serogroups in Drinking Water

**Helen Y. Buse [1,*], John S. Hall [1], Gary L. Hunter [2], and James A. Goodrich [1]**

[1] US Environmental Protection Agency (USEPA), Office of Research and Development (ORD), Center for Environmental Solutions & Emergency Response (CESER), Homeland Security and Materials Management Division, Cincinnati, OH; buse.helen@epa.gov (HYB), hall.john@epa.gov (JSH), goodrich.james@epa.gov (JAG)

[2] Black & Veatch; huntergl@bv.com (GLH)

* Correspondence: buse.helen@epa.gov (HYB)

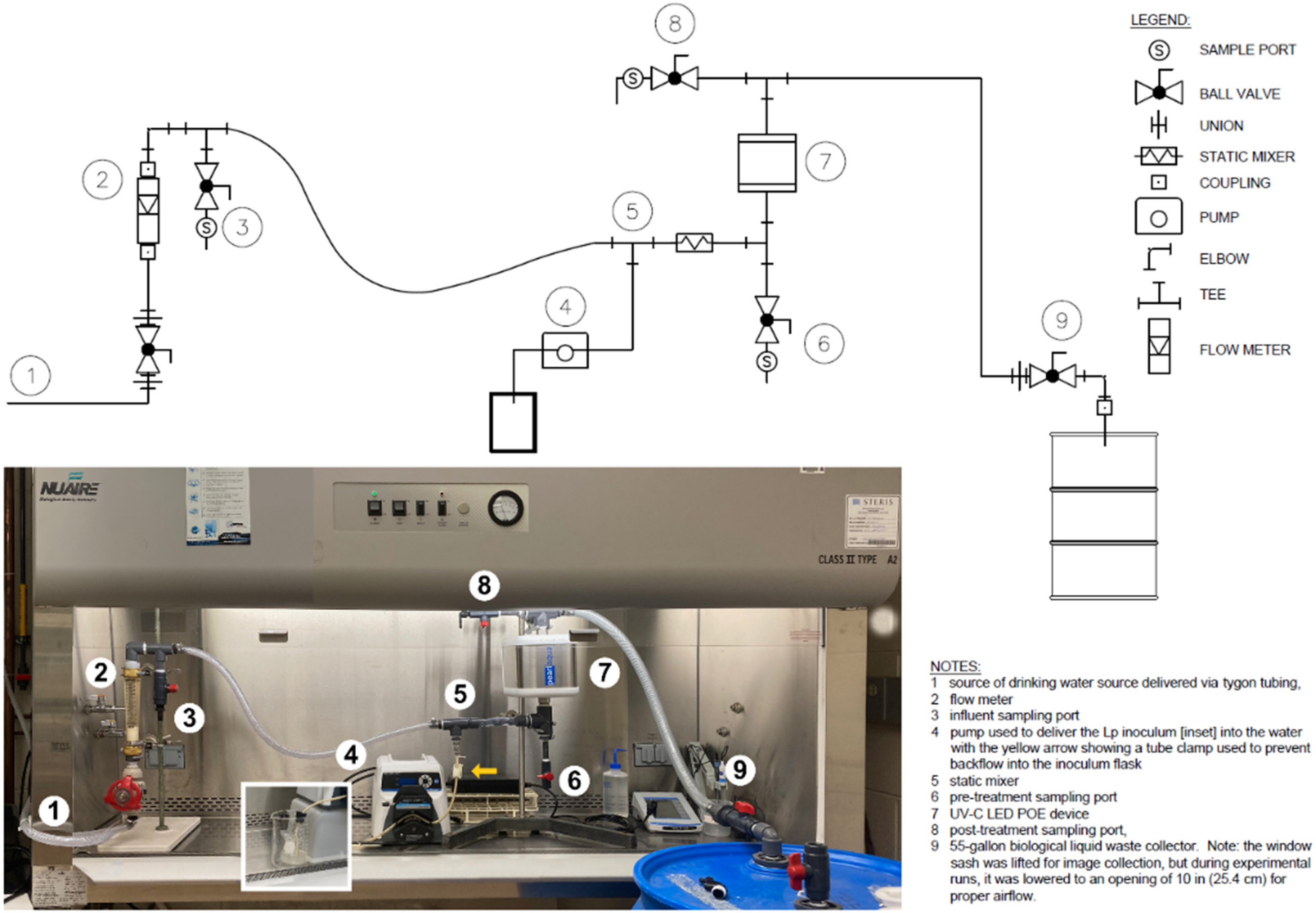


**Figure S1**. Schematic and image of the UV-C LED POE device test set-up.

**Table S1.** CFU levels of Lp strains after UV-C LED collimated beam exposure.

| Fluence | sg1 | | | sg1 DW | | | sg4 | | | sg6 | | |
|---|---|---|---|---|---|---|---|---|---|---|---|---|
| ($mJ/cm^2$) | 255nm | 265nm | 280nm | 255nm | 265nm | 280nm | 255nm | 265nm | 280nm | 255nm | 265nm | 280nm |
| 0 | 6.4 ± 0.1 | 6.4 ± 0.1 | 6.4 ± 0.1 | 6.8 ± 0.1 | 6.8 ± 0.1 | 6.8 ± 0.1 | 6.4 ± 0.2 | 6.4 ± 0.2 | 6.4 ± 0.2 | 6.5 ± 0.1 | 6.5 ± 0.1 | 6.5 ± 0.1 |
| 0.5 | 4.8 ± 0.1 | 6.2 ± 0.02 | 6.3 ± 0.1 | 4.4 ± 0.1 | 5.6 ± 0.1 | 6.4 ± 0.1 | 4.6 ± 0.3 | 6.1 ± 0.1 | 6.5 ± 0.04 | 4.7 ± 0.2 | 6.0 ± 0.04 | 6.4 ± 0.03 |
| 1 | 3.9 ± 0.4 | 5.6 ± 0.03 | 6.1 ± 0.01 | 2.6 ± 0.4 | 4.9 ± 0.2 | 6.1 ± 0.1 | 3.1 ± 0.3 | 5.4 ± 0.1 | 6.4 ± 0.1 | 2.9 ± 0.2 | 5.4 ± 0.1 | 6.1 ± 0.2 |
| 2 | 0.9 ± 0.2 | 5.1 ± 0.1 | 6.0 ± 0.1 | 1.7 ± 0.3 | 3.7 ± 0.2 | 5.4 ± 0.1 | nd | 4.8 ± 0.1 | 6.1 ± 0.1 | nd | 4.8 ± 0.1 | 5.7 ± 0.03 |
| 5 | nd | 3.9 ± 0.1 | 5.0 ± 0.01 | nd | 1.2 ± 0.1 | 4.5 ± 0.1 | nd | 3.7 ± 0.2 | 5.2 ± 0.04 | nd | 1.5 ± 0.4 | 4.3 ± 0.2 |
| 10 | nd | 0.9 ± 0.3 | 4.4 ± 0.1 | nd | 0.8 ± 0.2 | 1.6 ± 0.2 | nd | nd | 4.7 ± 0.4 | nd | nd | 3.4 ± 0.05 |
| 16 | - | - | 3.3 ± 0.1 | - | - | 1.3 ± 0.1 | - | - | 3.2 ± 0.1 | - | - | 1.4 ± 0.4 |
| 34 | - | - | 0.7 ± 0.0 | - | - | nd | - | - | nd | - | - | nd |

Data (mean $log_{10}$ ± standard deviation CFU/mL) are representative of three replicates for each fluence and wavelength.
-, not performed; nd, not detected (below LOD)

**Table S2.** CFU levels pre and post UV-C LED POE treatment.

| | | Lp strains tested | | | |
|---|---|---|---|---|---|
| | | sg1 | sg1 DW | sg4 | sg6 |
| Lp | pre | 5.9 ± 0.1 | 6.3 ± 0.1 | 5.5 ± 0.8 | 6.1 ± 0.2 |
| | post | 2.4 ± 0.5 | 3.0 ± 0.2 | 1.9 ± 0.8 | 1.1 ± 0.5 |
| | log reduction[1] | 3.5 ± 0.2 | 3.3 ± 0.1 | 3.6 ± 0.6 | 5.0 ± 0.3 |
| HPC | pre | 3.0 ± 0.2 | 3.5 ± 0.1 | 3.7 ± 0.2 | 2.9 ± 0.1 |
| | post | 1.8 ± 0.6 | 2.6 ± 0.4 | 1.9 ± 0.4 | 1.0 ± 0.0 |
| | log reduction[1] | 1.1 ± 0.3 | 0.9 ± 0.2 | 1.8 ± 0.2 | 1.9 ± 0.1 |

Data (mean $log_{10}$ ± standard deviation CFU/mL) are representative of five replicates for each strain.
[1]$SD_{LR}$ values are reported here with log reductions as described in Materials and Methods Section 2.6.
